# Beyond the Segmental Code: Anatomic Diversity and Surgical Pathways in Left Atrial Appendage Isomerism with the {A,L,I} Combination

**DOI:** 10.3390/diagnostics16213484

**Published:** 2026-10-24

**Authors:** Şerif Şerifoğlu, Fatma Sevinç Şengül, Mustafa Nalbant, Buse Günyel, Pelin Ayyıldız, Okan Yıldız, Selman Gökalp, Aysel Türkvatan Cansever, Sertaç Haydin, Alper Güzeltaş

**Affiliations:** 1 Department of Pediatric Cardiology, Mehmet Akif Ersoy Thoracic and Cardiovascular Surgery Training and Research Hospital, University of Health Sciences, Istanbul 34303, Türkiye; 2 Department of Cardiovascular Surgery, Mehmet Akif Ersoy Thoracic and Cardiovascular Surgery Training and Research Hospital, University of Health Sciences, Istanbul 34303, Türkiye; 3 Department of Radiology, Mehmet Akif Ersoy Thoracic and Cardiovascular Surgery Training and Research Hospital, University of Health Sciences, Istanbul 34303, Türkiye

**Keywords:** left atrial appendage isomerism, heterotaxy, situs ambiguus, segmental analysis, biventricular repair, pulmonary venous connections, congenital heart disease

## Abstract

**Background/Objectives:** Left atrial appendage isomerism (LAAI) is associated with substantial variation in venous connections and intracardiac anatomy. A subgroup of LAAI patients has L-loop ventricular topology together with a particular great artery relationship—a combination that corresponds historically to Van Praagh’s {A,L,I} segmental notation—but this subgroup has not been separately characterized. We aimed to describe the anatomical spectrum, rhythm and conduction abnormalities, biventricular repair eligibility, and surgical pathways in this subgroup. **Methods:** This single-center retrospective study included 15 patients with LAAI, L-loop ventricular topology, and a specific great artery relationship (aorta left-sided, pulmonary artery right-sided)—a combination corresponding historically to Van Praagh’s {A,L,I} notation—evaluated between January 2010 and December 2025. Diagnosis was confirmed independently by echocardiography and cardiac computed tomography in all patients. Anatomical suitability for biventricular repair was determined by multidisciplinary consensus. **Results:** Median age at presentation was 3.0 months, and median follow-up was 31.0 months. An interrupted inferior vena cava was present in all patients. The great artery relationship was aorta left-posterior/pulmonary artery right-anterior in 12 patients, aorta left-anterior/pulmonary artery right-posterior in 2, and side-by-side in 1. Ventriculoarterial connection was concordant in 8 patients, double-outlet right ventricle in 6, and double-outlet left ventricle in 1. A common atrioventricular valve was present in 7 patients. Right ventricular outflow tract obstruction was present in 7 patients. Arrhythmia was documented in 6 patients, and 2 required permanent pacemaker implantation. Ten patients (66.7%) were considered anatomically eligible for biventricular repair; repair had been completed in 4, 2 had received interim palliation, and 4 remained awaiting surgery. The remaining 4 patients followed a single-ventricle pathway. One death was confirmed, and 2 patients were lost to follow-up. **Conclusions:** Within LAAI, the coexistence of L-loop ventricular topology and a specific great artery relationship was associated with ventriculoarterial concordance in most patients, which may support biventricular repair. Anatomical eligibility for repair, however, did not always translate into completed repair. Larger, multicenter studies are needed to confirm these findings.

## 1. Introduction

**Figure 1:**
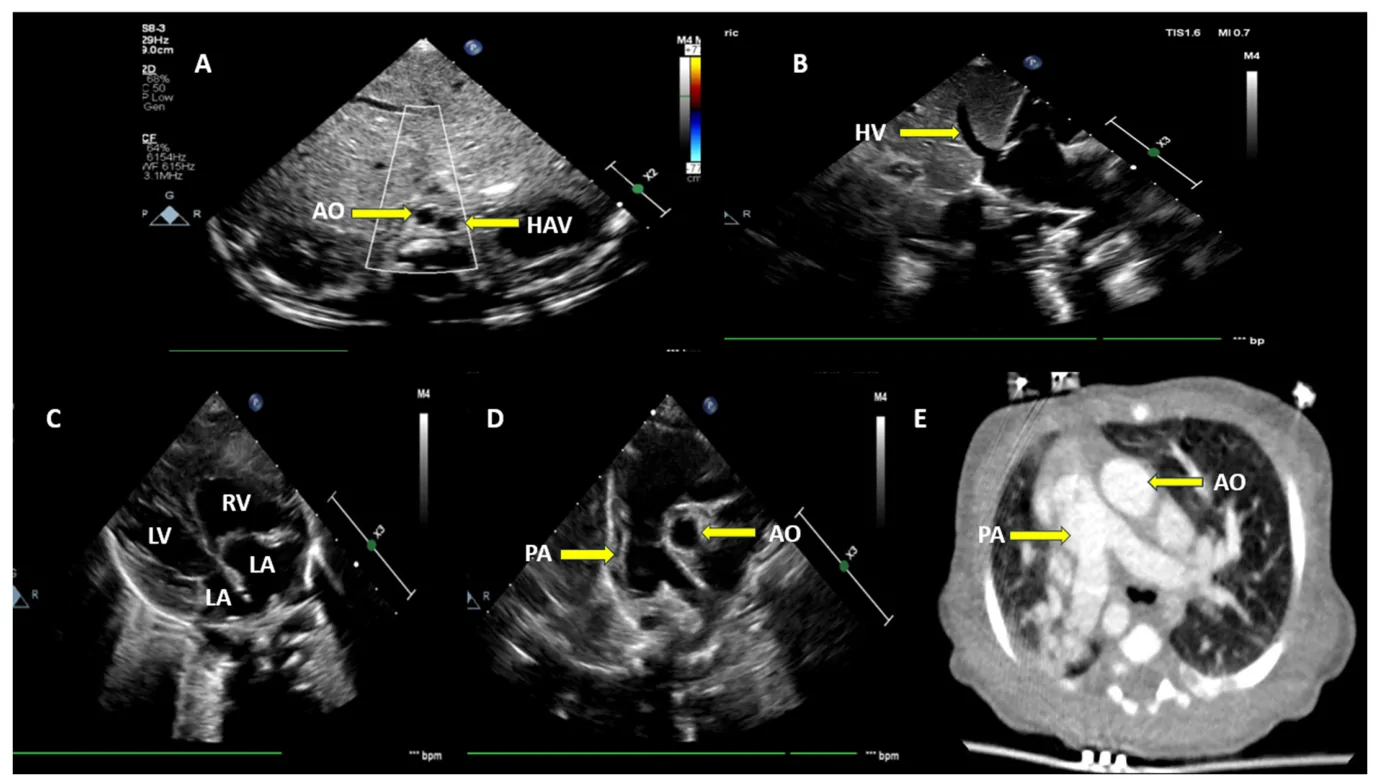
(**A**) Subcostal short-axis echocardiographic view showing the aorta (AO) and the hemiazygos vein (arrows), with the aorta positioned anteriorly and the hemiazygos vein (HAV) positioned posteriorly, a finding consistent with interruption of the inferior vena cava and a marker of left isomerism. (**B**) Subcostal long-axis echocardiographic view showing the hepatic vein (HV) draining separately into the right-sided atrium. (**C**) Apical four-chamber echocardiographic view showing an L-loop ventricular relationship, with the left ventricle (LV) positioned on the right and the right ventricle (RV) positioned on the left. LA:left atrium (**D**) Parasternal short-axis echocardiographic view showing an inversus relationship of the great arteries, with the pulmonary artery (PA) positioned to the right and the aorta (AO) to the left. (**E**) Axial CT image confirming the inversus relationship of the great arteries seen on echocardiography, with the PA positioned to the right and the aorta to the left.

When the arrangement of the thoracic and abdominal organs cannot be classified as situs solitus or situs inversus, the condition is considered situs ambiguus [1,2]. In Van Praagh’s segmental notation, atrial situs, ventricular loop, and the great artery relationship are coded with sequential letters; the letter “A” denotes situs ambiguus and encompasses both types of isomerism [3]. Sequential segmental analysis (SSA), developed by Anderson and colleagues, instead defines atrial appendage morphology, ventricular topology, and the great artery relationship separately and precisely [4,5]. (Figure 1A,C–E).

**Figure 2:**
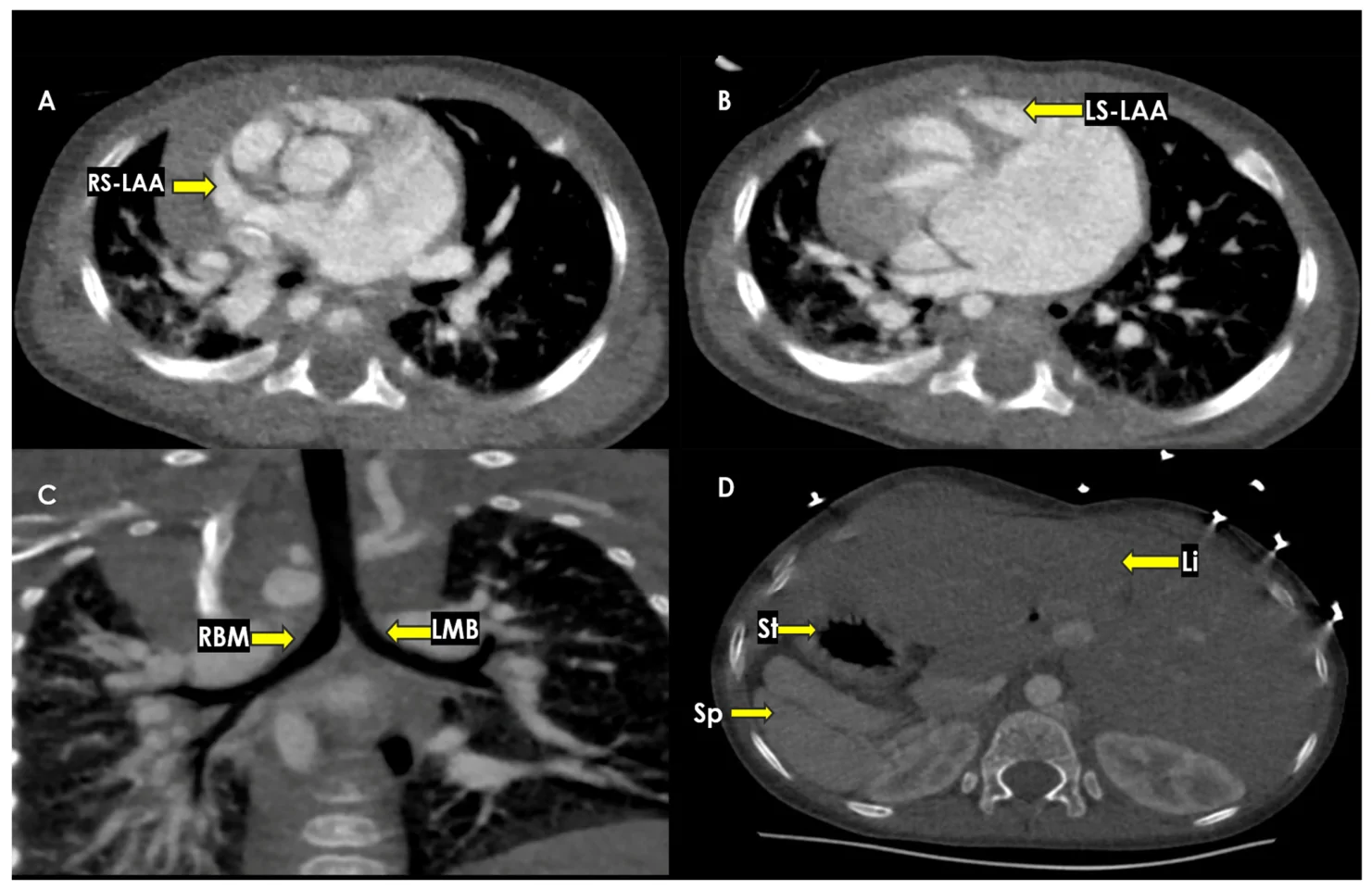
Axial and coronal Computed Tomography (CT) images demonstrating the morphological features of left atrial appendage isomerism (LAAI). (**A**,**B**) Axial CT slices showing bilateral morphologically left atrial appendages; (**A**) right-sided morphologically left atrial appendage (RS-LAA) and (**B**) left-sided morphologically left atrial appendage (LS-LAA). (**C**) Coronal CT image showing bilateral long, hyparterial mainstem bronchi (RBM: right main bronchus, LMB: left main bronchus), consistent with bronchial isomerism. (**D**) Abdominal axial CT slice showing midline liver (Li), right-sided stomach (St), and multiple spleens/polysplenia (Sp) in the right upper quadrant.

The reason for this lies in the fact that, in Van Praagh’s era, atrial appendage morphology could only be distinguished by pathological examination or surgical inspection. Modern imaging now allows independent and definitive confirmation of appendage morphology [1]. The (A,L,I) combination denotes L-loop ventricular topology together with a specific spatial relationship of the great arteries [3]; only about one third of patients with left atrial appendage isomerism (LAAI) have this topology [6]. This study examined this specific subgroup rather than the full LAAI population; left isomerism is characterized by bilateral bilobed lungs with a bronchial branching pattern consistent with left isomerism, polysplenia in most cases, interrupted inferior vena cava, and variable intracardiac malformations [2,7] (Figure 1B and Figure 2A–D).

Cardiac anatomy in this patient group differs from other forms of congenital heart disease because of the variety of systemic and pulmonary venous return anomalies involved. Bilateral superior vena cava, azygos or hemiazygos continuation, variation in hepatic venous drainage, and distribution of the pulmonary veins to both morphological left atria underlie this diversity [6,7]. Because the sinus node is hypoplastic or abnormally positioned, congenital or postoperative complete atrioventricular block has been reported in 7 to 20% of patients across large series [8,9,10]. Permanent pacemaker requirements range from 12 to 39% and stand out as a determinant of long-term morbidity [7,11,12]. Surgical outcomes vary considerably between patients; long-term series have reported survival rates of 63 to 87% in left isomerism, with mortality higher among those who undergo single-ventricle palliation [7,11,12,13].

**Figure 3:**
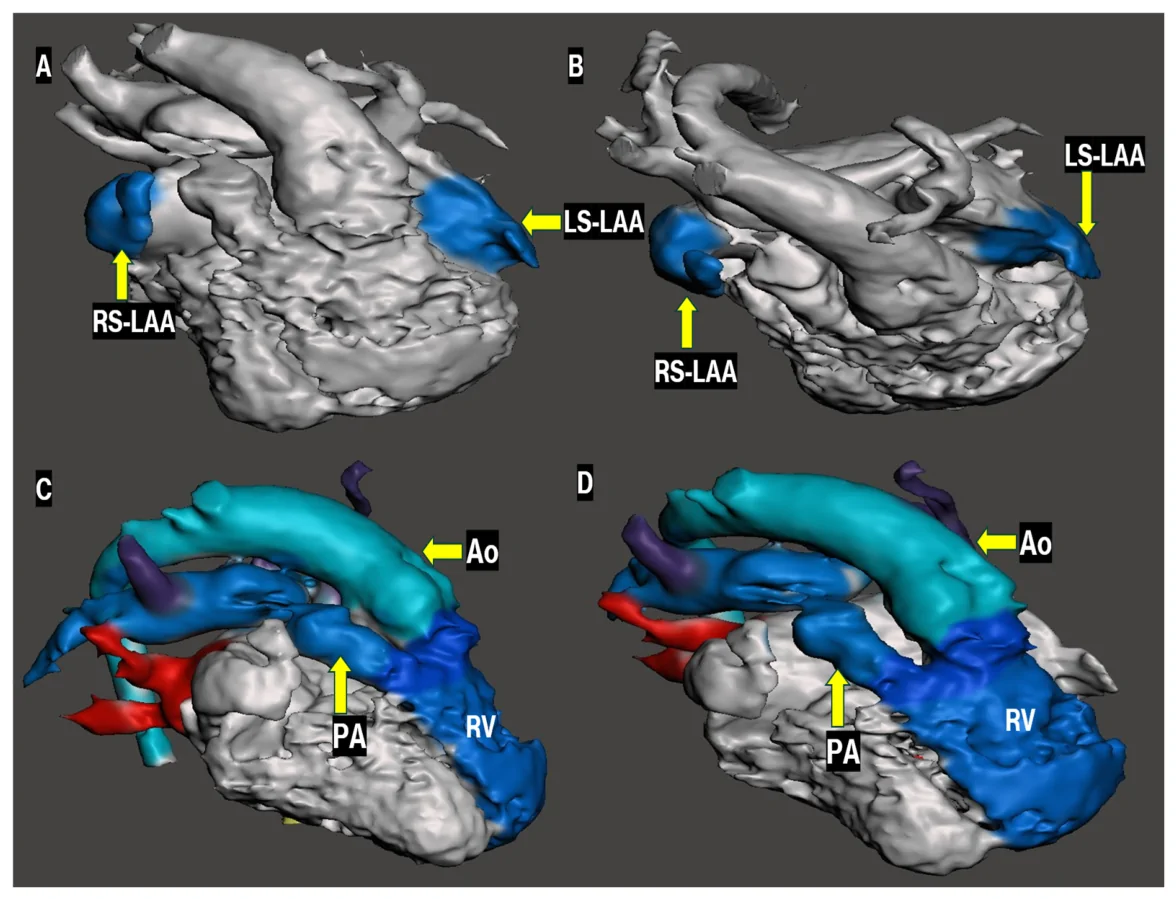
Three-dimensional cardiac reconstruction from computed tomography in one patient (patient 5), used for preoperative surgical planning. (**A**) Anterior view showing bilateral atrial appendages, both with left-sided morphology: the right-sided left atrial appendage (RS-LAA) and the left-sided left atrial appendage (LS-LAA). (**B**) Superior view of the same structures. (**C**) Anterior-right view showing the aorta (Ao) and pulmonary artery (PA) arising above the right ventricle (RV) (aorta left-anterior, pulmonary artery right-posterior). (**D**) Anterior view of the same structures. Ao, aorta; LS-LAA, left-sided left atrial appendage; PA, pulmonary artery; RS-LAA, right-sided left atrial appendage; RV, right ventricle.

Most published series have examined right and left isomerism together [6,11,14,15] or reported on left isomerism as a whole [7]. Findings specific to the (A,L,I) combination therefore remain difficult to compare. In this study, we retrospectively reviewed the clinical and anatomical characteristics and treatment outcomes of 15 patients followed at our center who met LAAI criteria and had L-loop topology together with a specific great artery relationship (aorta left-sided, pulmonary artery right-sided). These patients correspond historically to Van Praagh’s (A,L,I) segmental combination; LAAI patients with D-loop ventricular topology were not included in this study (Figure 3A–D).

## 2. Materials and Methods

### 2.1. Study Design and Patient Selection

This single-center retrospective observational study was conducted at Istanbul Mehmet Akif Ersoy Thoracic and Cardiovascular Surgery Training and Research Hospital. The study was approved by the local ethics committee (decision no. 2026.02-07; 24 February 2026), which waived the requirement for individual informed consent because of the retrospective design.

The institutional congenital heart disease database was retrospectively searched for patients evaluated between January 2010 and December 2025. The inclusion criterion was a diagnosis of left atrial appendage isomerism (LAAI) confirmed independently by echocardiography and computed tomography. LAAI was defined primarily by bilateral morphological left atrial appendages, supported by at least one of the following confirmatory findings: bilateral bilobed lungs with a bronchial branching pattern consistent with left isomerism, interrupted inferior vena cava, and/or polysplenia. This study examined not the full LAAI population but the subgroup with L-loop ventricular topology together with a specific great artery relationship (aorta left-sided, pulmonary artery right-sided), a combination that corresponds historically to Van Praagh’s (A,L,I) segmental notation. LAAI patients with D-loop ventricular topology were not included in this study. Patients with insufficient echocardiographic or computed tomographic data to complete segmental analysis were excluded.

### 2.2. Definitions and Terminology

Cardiac segmental anatomy was defined using the sequential segmental analysis (SSA) framework described by Anderson and colleagues [4,5]. Noting that this patient group corresponds historically to Van Praagh’s (A,L,I) notation, the corresponding terms from both systems were provided together: isomerism of the left atrial appendages (rather than “A”) and L-loop ventricular topology (rather than “L”) [3]. Ventricular topology is expressed in both systems: D-loop corresponds to right-hand ventricular topology, and L-loop corresponds to left-hand ventricular topology [1]. Heterotaxy terminology followed the nomenclature proposed by Jacobs et al. [1]. Because both atria were morphological left atria in all patients, the terms “right-sided atrium” and “left-sided atrium” are used throughout this report solely to indicate the spatial position of the morphological left atria; these terms do not imply the presence of a morphological right atrium.

Cardiac position was classified as dextrocardia, levocardia, or mesocardia based on the location of the ventricular mass within the thorax; apex direction was recorded separately and independently. In our patients, the term “inversus” referred to an arrangement in which the aorta is left-sided and the pulmonary artery is right-sided. This definition is independent of the type of ventriculo-arterial connection (concordant, DORV, or DOLV). In addition to this spatial relationship, the anterior-posterior relationship between the aorta and pulmonary artery was assessed separately and is reported in detail in the Results. Pulmonary venous return was described according to the spatial position of the receiving atrium rather than using conventional partial anomalous pulmonary venous drainage/total anomalous pulmonary venous drainage (PAPVD/TAPVD) terminology, given the absence of a morphological right atrium. Superior vena cava (SVC) laterality was defined by the vessel’s origin and course rather than by the chamber into which it drained; an SVC draining to the contralateral atrium therefore reflects a crossover in venous course rather than an inconsistency in naming. “Septum primum malposition” refers to displacement of the septum primum relative to its expected position at the atrioventricular junction.

Because both atrial chambers have the same (left) morphology, one chamber necessarily connects concordantly to its ventricle while the other connects discordantly, whenever the connection is biventricular. This arrangement is termed “mixed” in the SSA framework, regardless of whether the atrioventricular valve is common or separate [5].

### 2.3. Data Collection

Clinical, imaging, procedural, and follow-up data were retrospectively extracted from the institutional records. Anatomical data were compiled from echocardiographic records; in all patients (15/15), the diagnosis was supported by cardiac computed tomography (CT), allowing detailed assessment of segmental and venous anatomy. In one patient (Patient 7), CT images had been obtained at an outside institution prior to referral and were subsequently retrieved and included in the analysis.

Demographic and baseline clinical variables included sex, age, and body weight at presentation, peripheral oxygen saturation at presentation, and duration of follow-up. Follow-up duration was calculated from the first evaluation at our center to the last documented clinical contact or death.

Anatomical variables cardiac position and apex direction; type of inferior vena cava (IVC) continuation; SVC anatomy and drainage sites; hepatic venous drainage; pulmonary venous drainage pattern; great artery relationship; ventriculo-arterial relationship; atrial septal defect (ASD) type; atrioventricular valve type (common/separate) and ventricular septal defect/atrioventricular septal defect (VSD/AVSD) topography; degree of atrioventricular valve regurgitation; aortic arch anomalies; level and severity of right and left ventricular outflow tract obstruction (RVOTO/LVOTO); ventricular balance; abdominal situs; and other findings such as septum primum malposition and pulmonary hypertension.

Clinical and outcome variables included rhythm and conduction disturbances, their timing in relation to surgery, permanent pacemaker implantation, intended surgical pathway, procedures performed, follow-up status, and confirmed death during follow-up. Rhythm and conduction disturbances were identified from the available electrocardiographic and clinical records. At the final data cutoff, patients were categorized as being under active follow-up, lost to follow-up, or deceased.

### 2.4. Surgical Pathway and Assessment for Biventricular Repair

Patients were first classified according to the intended ventricular pathway as biventricular, single-ventricle, or not evaluable. Patients assigned to the biventricular pathway were further categorized as having undergone definitive biventricular repair, having received interim palliation before planned repair, or awaiting planned surgery. Patients assigned to the single-ventricle pathway were categorized according to the staged palliation performed. Individual surgical and catheter-based procedures were recorded separately.

Anatomical suitability for biventricular repair was determined by multidisciplinary consensus between the pediatric cardiology and congenital cardiac surgery teams. The assessment was based on ventricular balance, the location and routability of the ventricular septal defect, ventriculoarterial connection, atrioventricular valve morphology, and pulmonary venous anatomy as demonstrated by echocardiography and cardiac computed tomography. When intraoperative findings provided additional anatomical information, these findings were incorporated into the final classification. Patients without a documented multidisciplinary assessment were classified as not evaluable.

### 2.5. Statistical Analysis

Because of the small sample size and the descriptive nature of the study, the analysis was limited to descriptive statistics, and no inferential hypothesis testing was performed. Continuous variables were summarized as median, interquartile range, and range. Categorical variables were reported as n/N (%), with the relevant denominator specified when an analysis applied only to a subgroup or when data were unavailable. Statistical analyses were performed using IBM SPSS Statistics for Windows, version 22.0 (IBM Corp., Armonk, NY, USA).

## 3. Results

### 3.1. Demographic and Clinical Characteristics

The cohort comprised 15 patients, including 8 females (53.3%) and 7 males (46.7%). The median age at presentation was 3.0 months (IQR, 1.0–14.0; range, 0.2–84.0 months), and the median body weight was 4.1 kg (IQR, 3.3–8.5; range, 2.9–20.0 kg). Median peripheral oxygen saturation was 88% (IQR,82–91; range, 77–98%), and median follow-up was 31.0 months (IQR, 6.0–45.0; range, 3–96 months) (Table 1).

Clinical presentation varied in line with the wide age range at diagnosis. Four patients (26.7%) were diagnosed prenatally and followed from fetal life. Diagnosis followed evaluation for a murmur in three patients and evaluation for respiratory distress, tachypnea, or feeding difficulty in three others; this second group included one preterm infant intubated at birth for respiratory failure. In two patients, diagnosis was incidental to evaluation for an unrelated problem (cardiomegaly identified during work-up for fever; imaging obtained after trauma in the oldest patient in the cohort, at 84 months of age). The remaining two patients were referred for elective surgical evaluation with no symptoms other than reduced oxygen saturation.

### 3.2. Systemic and Pulmonary Venous Anatomy, Abdominal Situs

Cardiac position was dextrocardia in 11 patients (73.3%), levocardia in 3 (20.0%), and mesocardia in 1 (6.7%); apex direction was concordant with cardiac position in all patients. Interrupted inferior vena cava was present in all patients, with hemiazygos continuation in 11 and azygos continuation in 4 (Table 2(A)).

Superior vena cava anatomy was bilateral in 11 patients (73.3%), left-sided only in 3 (20.0%), and right-sided only in 1 (6.7%). Among the 12 patients with a right SVC, drainage was to the coronary sinus in 6 (50.0%), to the right-sided atrium in 5 (41.7%), and to the left-sided atrium in 1 (8.3%). Among the 14 patients with a left SVC, drainage was predominantly to the left-sided atrium (12 patients, 85.7%), and less commonly to the right-sided atrium (1 patient, 7.1%) or the coronary sinus (1 patient, 7.1%) (Table 2(A)).

Hepatic venous drainage was to the right-sided atrium in 7 patients (46.7%) and to the left-sided atrium in 7 (46.7%); in 1 patient (6.7%), the right hepatic vein drained to the right-sided atrium and the left hepatic vein drained to the left-sided atrium (Table 2(A)).

Pulmonary venous drainage was to both atria in 7 patients (46.7%), to the right-sided atrium in 6 (40.0%), and to the left-sided atrium in 2 (13.3%). Right pulmonary veins drained to the right-sided atrium in 13 patients (86.7%) and to the left-sided atrium in 2 (13.3%). Left pulmonary veins drained to the left-sided atrium in 9 patients (60.0%) and to the right-sided atrium in 6 (40.0%) (Table 2(A)).

The great artery relationship was aorta left-posterior/pulmonary artery right-anterior in 12 patients (80.0%), aorta left-anterior/pulmonary artery right-posterior in 2 (13.3%), and a side-by-side configuration in 1 (6.7%) (Table 2(A)).

Tracheobronchial branching pattern and lung lobation were assessed in all patients; bilateral bilobed lungs and a bronchial branching pattern consistent with left isomerism were found in all 15 patients (100%) (Figure) (Table 2(A)).

The liver was midline in all patients (100%). Polysplenia was present in 12 patients (80.0%); one patient had a single, normally sized spleen, and splenic status could not be assessed in 2 patients because it fell outside the imaged sections. Gastric position was right-sided in 9 patients (60.0%) and left-sided in 4 (26.7%); it could not be assessed in 2 patients (Table 2(A)) (Supplementary Table S1).

### 3.3. Cardiac Morphology and Associated Findings

The ventriculo-arterial relationship was concordant in 8 patients (53.3%), DORV in 6 (40.0%), and DOLV in 1 (6.7%). The aortic arch was right-sided in 11 patients (73.3%), left-sided in 3 (20.0%), and anterior-posterior in 1 (6.7%); arch hypoplasia was present in 3 patients (20.0%) (Table 2(B)).

The atrial septum was intact in 4 patients (26.7%), while no patient had an intact ventricular septum (Table 2(B)).

Atrial septal defect type was primum in 5 patients (33.3%), absent in 4 (26.7%), combined primum and secundum in 3 (20.0%), high venous in 2 (13.3%), and secundum in 1 (6.7%) (Table 2(B)).

A common atrioventricular valve was present in 7 patients (46.7%) and separate mitral and tricuspid valves in 8 (53.3%); in one patient (Patient 9), this common valve structure occurred in the context of an unbalanced AVSD with a hypoplastic right ventricle. The atrioventricular connection remained biventricular and did not fall into the double-inlet ventricle category (Table 2(B)).

Among the 7 patients with a common AV valve, the interventricular communication was classified separately from isolated VSD: small-inlet type in 5 and large inlet-outlet type in 2. Among the 8 patients with separate valves, isolated VSD topography was as follows: perimembranous outlet (n = 1), perimembranous outlet with posterior malalignment (n = 1), outlet with subaortic extension (n = 1), inlet with perimembranous-outlet extension (n = 3), muscular-bordered inlet (n = 1), and inlet with subaortic extension (n = 1). No doubly committed juxta-arterial defect was identified (Table 2(B)).

For the right-sided component of the common AV valve, regurgitation was absent in 1 patient and mild in 6; for the left-sided component, it was absent in 2, mild in 4, and moderate in 1. Among patients with separate valves, tricuspid regurgitation was absent in 3, mild in 3, mild-to-moderate in 1, and moderate in 1; mitral regurgitation was absent in 7 and moderate in 1.

Right ventricular outflow tract obstruction (RVOTO) was present in 7 patients (46.7%): at the valvular level, mild in 1 and severe in 1; at the subvalvular level, moderate in 2 and severe in 3. Left ventricular outflow tract obstruction (LVOTO) was present in 2 patients (13.3%), both subvalvular and mild; one of these patients (Patient 11) also had severe subvalvular RVOTO, with both obstructions attributed to restrictive tissue. No patient had aortic stenosis. Among the 10 patients considered eligible for biventricular repair, ventricular balance was unambiguous in all cases; one patient (Patient 9) had unbalanced ventricles, and no patient had isolated left ventricular hypoplasia (Table 2(B)).

Other findings included septum primum malposition in 3 patients (20.0%) and pulmonary hypertension in 6 (40.0%) (Table 2(B)) (Supplementary Table S1).

### 3.4. Rhythm and Conduction Disturbances

Electrophysiological study was not performed in any patient in this cohort. Baseline rhythm at presentation was sinus in 12 patients (80.0%), ectopic atrial rhythm in 2 (13.3%), and congenital complete AV block in 1 (6.7%); these findings pertain to the time of presentation, and serial rhythm data over the course of follow-up were not systematically reviewed.

Rhythm or conduction abnormalities were documented in 6 patients (40.0%). One patient had congenital complete AV block, and another developed persistent complete AV block after surgery; both underwent permanent pacemaker implantation. A third patient developed transient postoperative complete AV block, with recovery of AV conduction and no need for permanent pacing. Other documented abnormalities included Wolff–Parkinson–White syndrome, supraventricular tachycardia, and focal atrial tachycardia, each occurring in one patient (Table 3).

### 3.5. Biventricular Repair Eligibility, Surgical Management, and Follow-Up

In all surgically treated patients, the atrial appendage morphology identified intraoperatively was in full agreement with the preoperative echocardiographic and CT assessment. No patient showed a deviation from the initial treatment intent (crossover between the biventricular and single-ventricle pathways).

**Figure 4:**
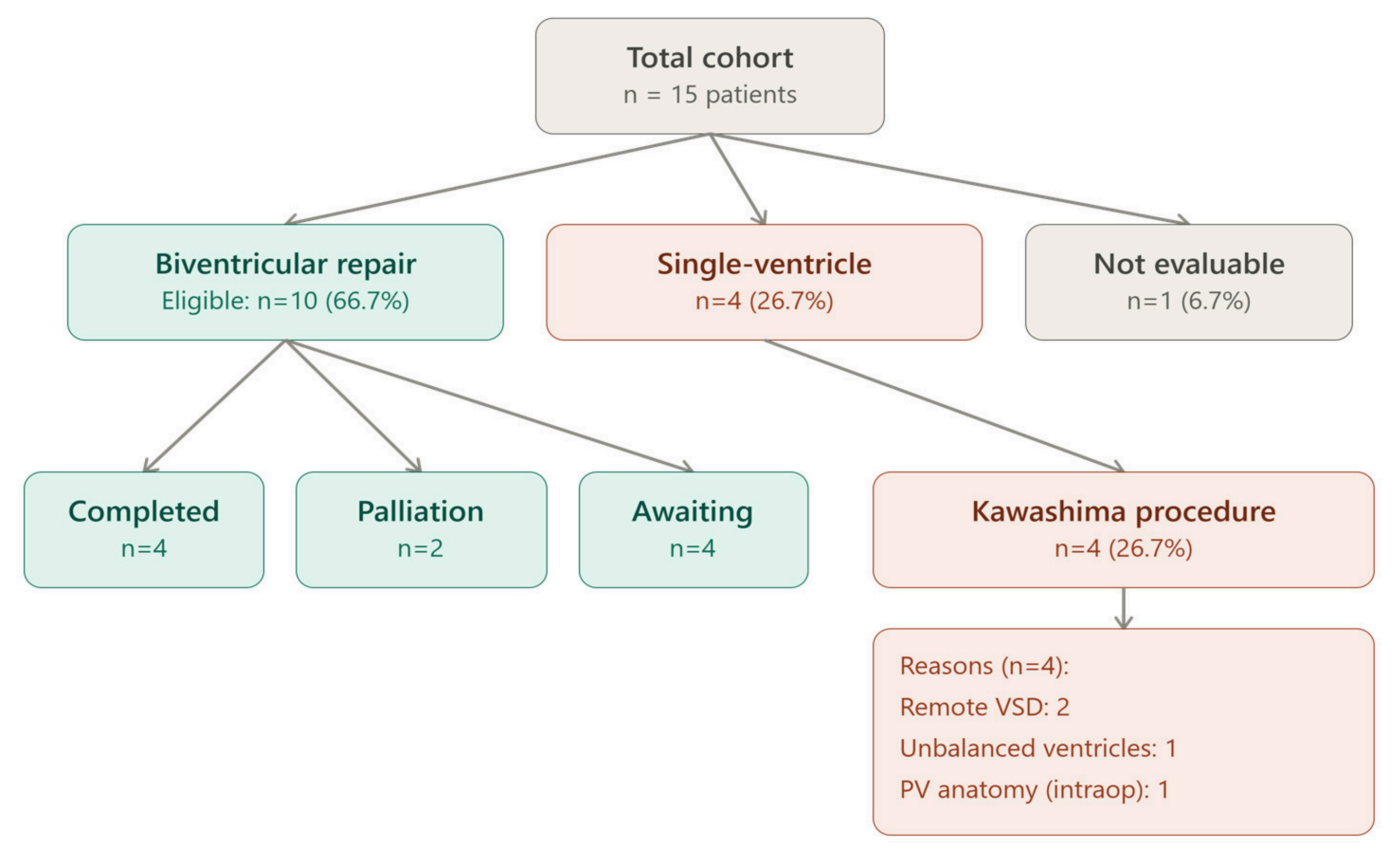
Classification of the cohort by biventricular repair eligibility. Ten patients (66.7%) were eligible; four underwent repair, two received interim palliation, and four were awaiting surgery. Four patients (26.7%) followed a single-ventricle pathway (Kawashima) due to remote VSD (*n* = 2), unbalanced ventricles (*n* = 1), or unfavorable pulmonary venous anatomy (*n* = 1). One patient (6.7%) was lost to follow-up before assessment.

On assessment by the pediatric cardiology and cardiac surgery team, 10 patients (66.7%) were considered anatomically eligible for biventricular repair. Of these, 4 (26.7%) underwent biventricular repair, 2 (13.3%) received interim palliation, and 4 (26.7%) were awaiting an appropriate time for surgery. The remaining 4 patients (26.7%) were directed to a single-ventricle pathway (Kawashima procedure). The reasons precluding biventricular repair in these patients were remote ventricular septal defect in 2, unbalanced ventricles in 1, and unfavorable pulmonary venous anatomy identified intraoperatively in 1. One patient (6.7%) could not be assessed because of loss to follow-up (Supplementary Table S1) (Table 3) (Figure 4).

At the end of follow-up, 12 patients (80.0%) were in active follow-up, 2 (13.3%) had been lost to follow-up, and 1 (6.7%) had died. Mortality during follow-up was limited to a single patient (1/15; 6.7%); this patient (Patient 9) underwent arch reconstruction with pulmonary artery banding, followed by a Kawashima procedure combined with atrioventricular valve repair, developed low cardiac output postoperatively requiring ECMO support, and died from an ECMO-related hemorrhagic complication. The survival status of the 2 patients lost to follow-up is unknown (Supplementary Table S1) (Table 3).

## 4. Discussion

In this single-center series of 15 patients with left atrial appendage isomerism and the {A,L,I} segmental combination, the same segmental code encompassed substantial variation in systemic and pulmonary venous connections, intracardiac anatomy, rhythm and conduction findings, and surgical pathways. Dextrocardia was present in 11 patients, a concordant ventriculoarterial connection in 8, and 10 of the 14 evaluable patients were considered anatomically suitable for biventricular repair. Because most previous studies have either combined right and left atrial appendage isomerism or examined left isomerism without stratification by segmental combination, direct comparison with our cohort remains limited [6,7,11,14,15].

One of the most consistent systemic venous findings was interruption of the inferior vena cava with azygos or hemiazygos continuation, which was present in all 15 patients. Gilljam et al. reported inferior vena cava interruption in 92% of 163 patients with left isomerism [7]. Although the proportion was numerically higher in our cohort, the small sample size and absence of a comparison group do not allow this finding to be attributed specifically to the {A,L,I} segmental combination.

Pulmonary venous connections were more heterogeneous. In 7 patients, pulmonary venous return was distributed between the right- and left-sided atrial chambers, whereas in the remaining patients it drained entirely to one atrial chamber. Kiran et al. reported partial anomalous pulmonary venous connection in 75.8% of patients with left isomerism in a computed tomography–based series [6]. Our classification, however, was based on the spatial position of the receiving atrial chamber rather than on conventional partial or total anomalous pulmonary venous connection categories. The two proportions therefore describe different anatomical classifications and should not be compared directly.

In this cohort, at least one arrhythmia was identified in 40.0% of patients (6/15); these included congenital and postoperative complete atrioventricular (AV) block, Wolff-Parkinson-White syndrome, and atrial tachyarrhythmias. The tendency toward arrhythmia in left isomerism has an anatomical basis in structural abnormalities of the conduction system. In their histopathological study of situs ambiguus cases, Dickinson et al. showed that the sinus node in left isomerism is hypoplastic and abnormally situated on the lateral atrial wall [8]. The same study described paired AV nodes connected by a conduction “sling” in cases with L-loop ventricular topology, a structural feature that provides an anatomical basis for AV conduction disturbances [8]. Because our entire cohort has L-loop topology, this anatomical prediction is directly relevant to our patient group.

Two permanent complete AV blocks were observed in our series, one congenital (6.7%) and one postoperative (6.7%). The congenital complete AV block rate in our series (1/15; 6.7%) is consistent with the 7% rate reported by Wren et al. for left isomerism [9]. Although the sample size limits inferential comparison, this finding is in line with the existing literature.

Permanent pacemaker implantation was performed in 2 patients (13.3%); both had permanent complete AV block. This rate falls at the lower end of the 12–39% pacemaker requirement range reported in large series [7,11].

Surgical management in this cohort showed a heterogeneous distribution: one quarter of patients were directed to a single-ventricle pathway (Kawashima procedure), one quarter to biventricular repair, and the remaining patients either received palliative intervention or were awaiting surgery. This distribution reflects the fact that surgical decision-making in left isomerism depends on the underlying anatomical substrate.

Eligibility for biventricular repair is one of the key features distinguishing left isomerism from right isomerism. In this cohort, two-thirds of patients (10/15; 66.7%) were found to be anatomically eligible for biventricular repair. Kim reported that approximately two-thirds of patients with left isomerism in general have anatomy suitable for biventricular repair [2]; our rate is consistent with this figure. In the series of 32 patients by Vodiskar et al., however, the proportion who actually underwent biventricular repair remained at 37.5% [16].

The high eligibility rate in our series may be related to features of this specific anatomical subgroup. Ventriculoarterial connection was concordant in just over half the cases (8/15, 53.3%); the remainder had double-outlet right ventricle (6/15) or double-outlet left ventricle (1/15). Ventriculoarterial concordance may facilitate biventricular repair by simplifying the surgical reconstruction of the outflow tracts. In the 4 cases not found eligible for biventricular repair, the obstacle was anatomical in nature: remote ventricular septal defect, unbalanced ventricles, or unfavorable pulmonary venous anatomy. In one of these cases, the obstacle was identified only intraoperatively, indicating that eligibility assessment is not limited to imaging alone.

This anatomical heterogeneity—spanning venous connections, intracardiac morphology, and surgical pathways—illustrates the difficulty of predicting biventricular repair eligibility from anatomical descriptors alone. Machine learning methods, capable of integrating multiple variables simultaneously, are increasingly contributing to decision-making in congenital heart disease [17]. Such tools could eventually support risk stratification and surgical planning in a subgroup this rare and anatomically diverse, although this would require datasets far larger and more multicentric than the present series.

Because biventricular repair in left isomerism requires complex atrial baffling, the risk of postoperative arrhythmia may be increased; the multiple suture lines created within the atrium raise the risk of injury to the conduction tissue. Vodiskar et al. reported postoperative arrhythmia in 66% of left isomerism cases undergoing biventricular repair [16]. In our series, the arrhythmia burden was 40.0%.

Mortality during follow-up was limited to a single patient (1/15; 6.7%). Because the survival status of the 2 patients (13.3%) lost to follow-up is unknown, the true mortality rate may be underestimated.

Left isomerism series in the literature show that mortality depends largely on the duration of follow-up and the surgical pathway [9,11,12,14]. In the series of 163 patients by Gilljam et al., overall survival declined to 56% at 28 years of follow-up, with 15-year survival of 48% in the single-ventricle group [7]. In the mixed cohort of 182 patients by Bhaskar et al., 18-year survival for left isomerism was reported as 74% [11]. These estimates are based on follow-up periods spanning decades, whereas our median follow-up is 31 months. The low mortality in our series may therefore be related to late events not yet having occurred, and does not reflect long-term survival.

## 5. Limitations

This study has several limitations. The single-center, retrospective design and small sample size (*n* = 15) do not allow for inferential statistical analysis; the findings can only be evaluated at a descriptive and hypothesis-generating level. The median follow-up of 31 months is insufficient for time-dependent outcomes such as late mortality and arrhythmia to become apparent; two patients were also lost to follow-up.

Coronary sinus anatomy was not routinely documented as a separate finding in our standard echocardiographic and CT reports; our imaging reports have traditionally focused on systemic venous connections at the level of the superior and inferior venae cavae. Ciliopathy was not systematically screened for in this cohort, and no patient had a clinical diagnosis. Three-dimensional modeling was used to support surgical planning only in selected patients and was not systematically recorded; an image from one such patient has been included in the figures. These gaps should be addressed in future prospective series with respect to complete sequential segmental analysis.

Anatomical classification was performed by consensus of the pediatric cardiology and cardiac surgery team, and our team does not include a formally trained clinical morphologist. Our center manages a high volume of complex congenital heart disease, including heterotaxy syndromes, which has allowed our team to gain experience with this anatomical spectrum.

Van Praagh’s three-letter notation belongs to an era that predates precise imaging, and it has been retained in this study only as a historical cross-reference; sequential segmental analysis should be the primary framework for classifying such patients. The hypothesis-generating value of this study does not derive from the {A,L,I} label but from the independently confirmed anatomical findings within this LAAI subgroup: the coexistence of L-loop ventricular topology and a specific great artery relationship was associated with ventriculoarterial concordance in approximately half the patients, which may facilitate biventricular repair.

## 6. Conclusions

This subgroup, historically defined using Van Praagh’s three-letter {A,L,I} notation, remains incomplete under that system because it does not directly specify atrial appendage morphology, nor does it define the full spatial relationship of the great arteries; it is more precisely and comprehensively classified using sequential segmental analysis, which allows definitive, imaging-based description of atrial, ventricular, and arterial morphology. Within left atrial appendage isomerism, the coexistence of L-loop ventricular topology and a specific great artery relationship (aorta left-sided, pulmonary artery right-sided) defines a distinct subgroup. In this subgroup, ventriculoarterial concordance was present in about half of the patients, which may favor biventricular repair. This anatomy can be identified early and accurately with echocardiography and computed tomography; three-dimensional CT can further support the assessment of biventricular eligibility. Because arrhythmia and conduction disturbances were also common in this group, long-term rhythm follow-up should be part of this evaluation. Larger, multicenter studies are needed to confirm these findings.

## Figures and Tables

**Table 1:** Baseline demographic and clinical characteristics of patients with left atrial appendage isomerism and the {A,L,I} segmental combination (*n* = 15).

Variable	*n* (%)
***Sex***	
Male	7 (46.7%)
Female	8 (53.3%)
***Clinical parameters at presentation, median (Q1–Q3; range)***	
Age (months)	3.0 (1.0–14.0; 0.2–84.0)
Body weight (kg)	4.1 (3.3–8.5; 2.9–20.0)
SpO_2_ (%)	88 (82–91; 77–98)
Follow-up duration (months)	31.0 (6.0–45.0; 3–96)

IQR, interquartile range; SpO_2_, peripheral oxygen saturation. Continuous variables are expressed as median (Q1–Q3 interquartile range; minimum–maximum range). Categorical variables are expressed as *n* (%).

**Table 2:** Anatomical findings in patients with left atrial appendage isomerism and the {A,L,I} segmental combination (*n* = 15). (**A**) Cardiac position and systemic and pulmonary venous anatomy (*n* = 15). Abdominal situs and tracheobronchial arrangement (*n* = 15). (**B**) Intracardiac morphology (*n* = 15).

**(A)**	
**Variable**	**n (%)**
***Cardiac position and apex direction***	
Dextrocardia (apex right-inferior)	11 (73.3%)
Levocardia (apex left-inferior)	3 (20.0%)
Mesocardia (apex right-inferior)	1 (6.7%)
***Inferior vena cava continuation***	
Hemiazygos continuation	11 (73.3%)
Azygos continuation	4 (26.7%)
***Superior vena cava (SVC) anatomy***	
Bilateral SVC	11 (73.3%)
Left SVC only	3 (20.0%)
Right SVC only	1 (6.7%)
***Right SVC drainage (n = 12) a***	
To right-sided atrium	5 (41.7%)
To coronary sinus	6 (50.0%)
To left-sided atrium	1 (8.3%)
***Left SVC drainage (n = 14) b***	
To left-sided atrium	12 (85.7%)
To right-sided atrium	1 (7.1%)
To coronary sinus	1 (7.1%)
***Hepatic vein drainage***	
To right-sided atrium	7 (46.7%)
To left-sided atrium	7 (46.7%)
Bilateral (right hepatic→RA, left hepatic→LA)	1 (6.7%)
***Pulmonary venous drainage pattern***	
To both atria	7 (46.7%)
To right-sided atrium	6 (40.0%)
To left-sided atrium	2 (13.3%)
***Right pulmonary veins***	
Draining to right-sided atrium	13 (86.7%)
Draining to left-sided atrium	2 (13.3%)
***Left pulmonary veins***	
Draining to left-sided atrium	9 (60.0%)
Draining to right-sided atrium	6 (40.0%)
***Great artery relationship***	
Aorta left-posterior/PA right-anterior	12 (80.0%)
Aorta left-anterior/PA right-posterior	2 (13.3%)
Side-by-side	1 (6.7%)
***Liver position***	
Midline	15 (100%)
***Splenic status***	
Polysplenia	12 (80.0%)
Single, normal spleen	1 (6.7%)
Not determinable	2 (13.3%)
***Gastric bubble position***	
Right-sided	9 (60.0%)
Left-sided	4 (26.7%)
Not determinable	2 (13.3%)
***Tracheobronchial arrangement***	
Bronchial pattern consistent with left isomerism	15 (100%)
***Lung lobation***	
Bilateral bilobed lungs	15 (100%)
(**B**)	
***Ventriculo-arterial relationship***	
Concordant	8 (53.3%)
Double outlet right ventricle (DORV)	6 (40.0%)
Double outlet left ventricle (DOLV)	1 (6.7%)
***Aortic arch***	
Right aortic arch	11 (73.3%)
Left aortic arch	3 (20.0%)
Anterior-posterior arch	1 (6.7%)
Arch hypoplasia	3 (20.0%)
***Atrial septal defect (ASD) type c***	
Primum	5 (33.3%)
Absent (intact atrial septum)	4 (26.7%)
Primum + Secundum	3 (20.0%)
High venous	2 (13.3%)
Secundum	1 (6.7%)
**Intact ventricular septum**	0 (0%)
***Atrioventricular valve morphology***	
Common atrioventricular valve	7 (46.7%)
Separate mitral and tricuspid valves	8 (53.3%)
Unbalanced AVSD with hypoplastic RV (Patient 9)	1 (6.7%)
***Interventricular communication—common AV valve (n = 7) d***	
Small-inlet type	5 (71.4%)
Large inlet-outlet type	2 (28.6%)
***Ventricular septal defect—separate valves (n = 8) d***	
Perimembranous outlet	1 (12.5%)
Perimembranous outlet, posterior malalignment	1 (12.5%)
Outlet, subaortic extension	1 (12.5%)
Inlet + perimembranous-outlet extension	3 (37.5%)
Inlet, muscular border	1 (12.5%)
Inlet, subaortic extension	1 (12.5%)
Doubly committed juxta-arterial defect	0 (0%)
***Right ventricular outflow tract obstruction (RVOTO)***	
None	8 (53.3%)
Valvular, mild	1 (6.7%)
Valvular, severe	1 (6.7%)
Subvalvular, moderate	2 (13.3%)
Subvalvular, severe	3 (20.0%)
***Left ventricular outflow tract obstruction (LVOTO)***	
None	13 (86.7%)
Subvalvular, mild	2 (13.3%)
Aortic stenosis	0 (0%)
***Ventricular balance***	
Balanced (unambiguous)	14 (93.3%)
Unbalanced (Patient 9)	1 (6.7%)
Borderline ventricles	0 (0%)
Isolated left ventricular hypoplasia	0 (0%)
***Other findings***	
Septum primum malposition	3 (20.0%)
Pulmonary hypertension	6 (40.0%)

PA, pulmonary artery; RA, right-sided morphological left atrium; LA, left-sided morphological left atrium; SVC, superior vena cava. a Right SVC drainage data available for 12 patients (those with right-sided or bilateral SVC). b Left SVC drainage data available for 14 patients (those with left-sided or bilateral SVC). SVC laterality is defined by vessel origin and course, not by the chamber of drainage; a left-sided SVC draining to the right-sided atrium (or vice versa) reflects a crossover in venous course. Apex direction was concordant with cardiac position in all patients. Abdominal situs was assessed on echocardiography and computed tomography. AV, atrioventricular; AVSD, atrioventricular septal defect; DOLV, double outlet left ventricle; DORV, double outlet right ventricle; RV, right ventricle. c Three patients with absent ASD were confirmed on review of echocardiographic records. d The interventricular communication associated with the common atrioventricular valve is classified separately from isolated ventricular septal defects; the two categories together account for all 15 patients (7 + 8). Atrioventricular valve regurgitation severity is reported in the Results and in the Supplementary Table S1. Given the isomeric atrial morphology present in all patients, the atrioventricular connection is necessarily half-concordant and half-discordant (“mixed” in the sequential segmental analysis framework) in patients with biventricular connections, irrespective of whether the atrioventricular valve is common or separate. Septum primum malposition refers to displacement of the septum primum relative to its expected position at the atrioventricular junction.

**Table 3:** Rhythm findings, clinical outcomes, and biventricular repair eligibility (*n* = 15).

Variable	n (%)
***Follow-up status***	
Active follow-up	12 (80.0%)
Lost to follow-up	2 (13.3%)
Deceased	1 (6.7%)
***Mortality***	
Confirmed mortality	1 (6.7%)
No mortality	12 (80.0%)
Unknown (lost to follow-up)	2 (13.3%)
***Baseline rhythm at presentation***	
Sinus rhythm	12 (80.0%)
Ectopic atrial rhythm	2 (13.3%)
Congenital complete AV block	1 (6.7%)
***Arrhythmia during follow-up***	
Any arrhythmia	6 (40.0%)
Congenital AV complete block	1 (6.7%)
Postoperative permanent AV complete block	1 (6.7%)
Postoperative transient AV complete block	1 (6.7%)
Wolff-Parkinson-White syndrome	1 (6.7%)
Supraventricular tachycardia	1 (6.7%)
Focal atrial tachycardia	1 (6.7%)
Pacemaker implantation	2 (13.3%)
***Biventricular repair eligibility***	
Eligible	10 (66.7%)
Repair performed	4 (26.7%)
Interim palliation	2 (13.3%)
Awaiting surgery	4 (26.7%)
Not eligible (single-ventricle pathway)	4 (26.7%)
Remote VSD	2
Unbalanced ventricles	1
Pulmonary venous anatomy (intraoperative)	1
Not evaluable (lost to follow-up)	1 (6.7%)

*AV, atrioventricular; VSD, ventricular septal defect.* Baseline rhythm reflects the rhythm documented at initial presentation; serial rhythm data over the course of follow-up were not systematically reviewed. Electrophysiological study was not performed in any patient. Both pacemaker recipients had permanent AV complete block (1 congenital, 1 postoperative). Postoperative transient AV complete block resolved without pacemaker implantation. Biventricular repair eligibility was determined by consensus of the pediatric cardiology and cardiac surgery team based on balanced ventricular size, ventriculoarterial relationship, and atrioventricular valve morphology.

## Data Availability

The data presented in this study are available on reasonable request from the corresponding author. The data are not publicly available due to privacy and ethical restrictions concerning patient medical records.

## Supplementary Materials

The following supporting information can be downloaded at: https://www.mdpi.com/article/10.3390/diagnostics16213484/s1, Table S1: Individual patient anatomy, interventions, and status at last follow-up (*n* = 15).
