# Supplementary material for: Beyond the Segmental Code: Anatomic Diversity and Surgical Pathways in Left Atrial Appendage Isomerism with the {A,L,I} Combination
Source: Diagnostics (Basel). 2026 Oct 24;16(21):3484. doi: 10.3390/diagnostics16213484 (PMC13761808; doi:10.3390/diagnostics16213484)
Supplement: Supplementary file 1 [file diagnostics-16-03484-s001.zip › diagnostics-4569693-supplementary.pdf]

Supplementary Table S1. Individual patient anatomy, interventions, and status at last follow-up (*n*=15)

Patient	Anatomical diagnosis	Intervention(s) / current plan	Status at last follow-up
1	Dextrocardia, apex right-inferior<br>Aorta left-posterior/PA right-anterior<br>Common AV valve, small-inlet type<br>Hepatic venous return to RAC<br>Pulmonary venous return to RAC	Planned ASD closure and hepatic venous rerouting<br>Awaiting surgery	Active follow-up
2	Dextrocardia, apex right-inferior<br>Aorta left-posterior/PA right-anterior<br>Common AV valve, small-inlet type<br>Hepatic venous return to LAC<br>Pulmonary venous return to RAC	AVSD repair	Active follow-up
3	Dextrocardia, apex right-inferior<br>Aorta left-posterior/PA right-anterior<br>Separate AV valves; perimembranous outlet VSD; ASD<br>Valvular RVOTO, mild; PH<br>Septum primum malposition<br>Ipsilateral pulmonary venous connections (right PVs→RAC; left PVs→LAC)	1. Pulmonary artery banding<br>2. Kawashima operation	Active follow-up
4	Mesocardia, apex right-inferior<br>Aorta left-anterior/PA right-posterior<br>Common AV valve, large inlet-outlet type; DORV<br>Subvalvular RVOTO, moderate<br>Hepatic venous return to RAC; right SVC to RAC<br>Ipsilateral pulmonary venous connections (right PVs→RAC; left PVs→LAC)	Modified BT shunt	Lost to follow-up
5	Levocardia, apex left-inferior<br>Aorta left-posterior/PA right-anterior<br>Separate AV valves, inlet VSD of muscular border; DORV; large ASD; PH<br>Bilateral SVCs; right SVC to RAC<br>Ipsilateral pulmonary venous connections (right PVs→RAC; left PVs→LAC)	1. Pulmonary artery banding<br>2. Kawashima operation	Active follow-up
6	Levocardia, apex left-inferior<br>Aorta left-anterior/PA right-posterior<br>Separate AV valves; perimembranous outlet VSD with posterior malalignment; mild aortic arch hypoplasia<br>Subvalvular LVOTO, mild<br>Septum primum malposition<br>Ipsilateral pulmonary venous connections (right PVs→RAC; left PVs→LAC)	Awaiting planned surgery; clinical follow-up	Active follow-up
7	Dextrocardia, apex right-inferior<br>Aorta left-posterior/PA right-anterior	1. Modified BT shunt<br>2. VSD closure, RVOT enlargement, and BT shunt takedown	Active follow-up

	Separate AV valves; outlet VSD, subaortic extension; DORV<br>Subvalvular RVOTO, moderate		
8	Dextrocardia, apex right-inferior<br>Aorta left-posterior/PA right-anterior<br>Separate AV valves; inlet + perimembranous-outlet VSD; DORV<br>Subvalvular RVOTO, severe<br>Hepatic venous return to RAC	Bilateral Kawashima, VSD enlargement, and atrial septectomy	Active follow-up
9	Dextrocardia, apex right-inferior<br>Aorta left-posterior/PA right-anterior<br>Common AV valve, large inlet-outlet type, unbalanced with hypoplastic right ventricle; DOLV<br>Aortic arch hypoplasia; PH<br>Hepatic venous return to RAC	1. Arch reconstruction and pulmonary artery banding<br>2. Kawashima operation and AV valve repair	Deceased (ECMO-related hemorrhage after low cardiac output)
10	Levocardia, apex left-inferior<br>Aorta left-posterior/PA right-anterior<br>Separate AV valves; inlet + perimembranous-outlet VSD; aortic coarctation; ASD; PH<br>Right SVC to RAC<br>Ipsilateral pulmonary venous connections (right PVs→RAC; left PVs→LAC)	1. Coarctation repair and pulmonary artery banding<br>2. VSD closure, pulmonary venous baffle to RAC, transannular patch, PTFE conduit PVR, and left-sided AV valve repair	Active follow-up
11	Dextrocardia, apex right-inferior<br>Aorta left-posterior/PA right-anterior<br>Common AV valve, small-inlet type<br>Subvalvular RVOTO, severe; subvalvular LVOTO, mild<br>Hepatic venous return to RAC<br>Pulmonary venous return to LAC	VSD closure, LVOTO relief, pulmonary stenosis relief, and pulmonary venous rerouting to RAC	Active follow-up
12	Dextrocardia, apex right-inferior<br>Side-by-side great arteries<br>Separate AV valves; inlet VSD, subaortic extension<br>Subvalvular RVOTO, severe<br>Hepatic venous return to RAC; right SVC to RAC	1. Right modified BT shunt (external center)<br>2. Left modified BT shunt (external center)	Lost to follow-up
13	Dextrocardia, apex right-inferior<br>Aorta left-posterior/PA right-anterior<br>Common AV valve, small-inlet type; PH<br>Ipsilateral pulmonary venous connections (right PVs→RAC; left PVs→LAC)	Pulmonary hypertension therapy initiated; awaiting surgery	Active follow-up
14	Levocardia, apex left-inferior<br>Aorta left-posterior/PA right-anterior<br>Common AV valve, small-inlet type (right-sided component regurgitation severe, left-sided mild)<br>Large PDA; large primum ASD; systemic PH<br>Hepatic venous return to RAC<br>Ipsilateral pulmonary venous connections (right PVs→RAC; left PVs→LAC)	PDA ligation	Active follow-up

15	Dextrocardia, apex right-inferior<br>Aorta left-posterior/PA right-anterior<br>Separate AV valves; inlet + perimembranous-outlet VSD<br>Valvular RVOTO, severe<br>Bilateral SVCs; hepatic venous return to LAC	No intervention; under surveillance	Active follow-up

RAC and LAC indicate the right-sided and left-sided atrial chambers, respectively, and refer only to spatial position; they do not imply morphologically right or left atria. In left atrial appendage isomerism, each atrial chamber has a morphologically left appendage.

Ipsilateral pulmonary venous connections indicate right pulmonary veins to RAC and left pulmonary veins to LAC. Apex direction was concordant with cardiac position in all patients.

Coronary sinus anatomy and tracheobronchial/lobation pattern are pending and will be added to this table once systematically reviewed.

ASD, atrial septal defect; AV, atrioventricular; AVSD, atrioventricular septal defect; BT, Blalock-Taussig; DOLV, double-outlet left ventricle; DORV, double-outlet right ventricle; ECMO, extracorporeal membrane oxygenation; LAC, left-sided atrial chamber; LVOTO, left ventricular outflow tract obstruction; PA, pulmonary artery; PDA, patent ductus arteriosus; PH, pulmonary hypertension; PTFE, polytetrafluoroethylene; PV, pulmonary vein; PVR, pulmonary valve replacement; RAC, right-sided atrial chamber; RVOT(O), right ventricular outflow tract (obstruction); SVC, superior vena cava; VSD, ventricular septal defect.
